# Supplementary material for: Beyond Epitaxy: Ion Implantation as a Tool for Orbital Engineering
Source: ACS Appl Electron Mater. 2025 Jul 29;7(16):7580–4. doi: 10.1021/acsaelm.5c00815 (PMC12392450; doi:10.1021/acsaelm.5c00815)
Supplement: Supplementary file 1 [file el5c00815_si_001.pdf]

Supplementary Material

Beyond Epitaxy: Ion Implantation as a Tool for Orbital Engineering

Andreas Herklotz[1,*], Jonathan R. Petrie[2], Thomas Z. Ward[3,*]

[1] Institute for Physics, Martin-Luther-University Halle-Wittenberg, 06120 Halle, Germany
[2] Materials Science and Technology Division, Oak Ridge National Laboratory, 37830 Oak Ridge, TN, USA
[3] Center for Nanophase Materials Sciences, Oak Ridge National Laboratory, 37830 Oak Ridge, TN, USA
[*] Corresponding Authors

Email: herklotza@gmail.com; wardtz@ornl.gov

SRIM calculations

SRIM-2013 calculations on an Au (15nm)/ LNO (12nm)/ STO heterostructure have been performed in order simulate the He ion distribution over the LNO film. The result for a calculations with a total of 4000 ion irradiation trajectories is shown below. The results show that the peak of the He ion concentration indeed lies within the LNO layer, with a slightly higher concentration near the surface.

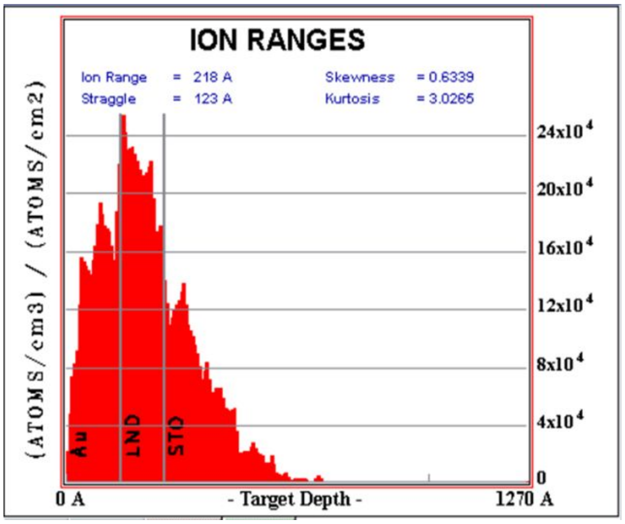

Figure S1. Perpendicular He ion distribution calculated with the *SRIM-2013* software package for the Au (15nm)/ LNO (12nm)/ STO heterostructure and an implantation energy of 4 keV.

XRD rocking curves

The figure below shows the rocking curves of the as-grown (black) and 1E16 He/cm^2 implanted (red) sample around the 103_{pc} film peak. No peak broadening is observed, indicating no major effect on the crystallographic film quality due to implantation-induced point defects.

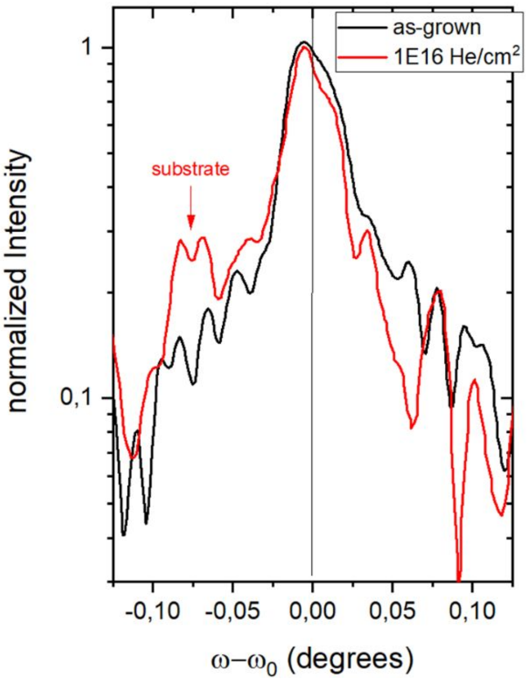

Figure S2. Rocking curves about the 103_{pc} film peaks, showing no evidence of peak broadening due to point defects or other crystallographic defects.

AFM topography after Au gold removal

The figure below shows the AFM topography image of the 1E16 He/cm2 film after the Au protective layer was removed. No residual gold particles and no surface modification due to the ion beam irradiation is observed. The LNO film surface is atomically smooth and shows the terrace-like surface from the treated STO substrate, essentially identical to that of the as-grown LNO film.

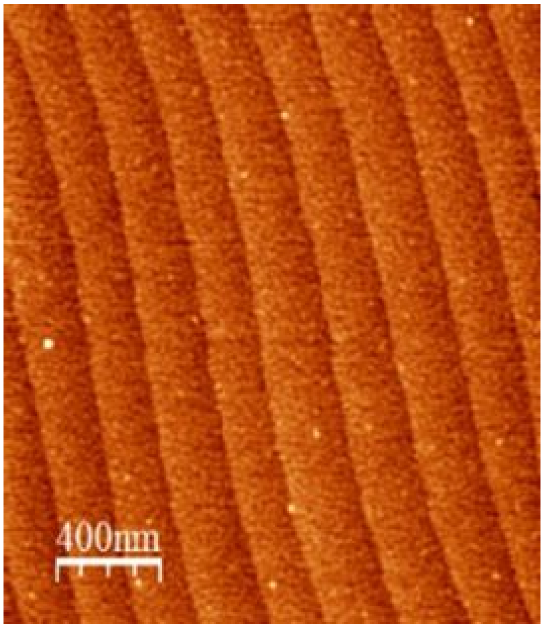

Figure S3. AFM topography image of the 1E16 He/cm^2 implanted film after removal of the Au protective layer.

Determination of d_z^2 occupancy using x-ray linear dichroism (XLD)

In order to determine the dz2 occupancy we follow method described by Wu et. al. [1] and Disa et al. [2]. The ratio of holes (unoccupied states) in the e_g orbitals is calculated through XLD sum rules as:

$$r = \frac{h_{z^2}}{h_{x^2-x^2}} = \frac{3I_c}{4I_{ab} - I_c}$$

where h_{z^2} is the hole occupancy number of the d_{z^2} orbitals, $h_{x^2-y^2}$ is the hole occupancy number of the $d_{x^2-y^2}$ orbitals, I_c is the X-ray absorption along the out-of-plane direction, and I_{ab} is the X-ray absorption along the in-plane direction.

There are two possible states in each e_g orbital, or $n_{z^2} = 2 - h_{z^2}$ and $n_{x^2-y^2} = 2 - h_{x^2-y^2}$, where n corresponds to the electron occupancy number and h corresponds to the electron occupancy number in each respective orbital. Adding the states of both orbitals, $n_{e_g} + h_{e_g} = 4$, where n_{e_g} is the total electron occupancy number and h_{e_g} is the total hole occupancy number. Using the prior assumption that $n_{e_g} = 1$, $h_{e_g} = 3 = h_{z^2} + h_{x^2-y^2}$ and the fraction d_{z^2} occupancy f(d_{z^2}) can be determined as:

$$f(d_{z^2}) = \frac{n_{z^2}}{n_{eg}} = n_{z^2} = 2 - h_{z^2} = 2 - \frac{3r}{1+r}$$

To determine the intensities, we took the average of different Pseudo-Voigt linear combinations of Gaussian and Lorentzian curves to fit the XLD data. The error was determined by the standard deviations of the fits. Errors for intensities were added in quadrature when used for determining orbital occupancy.

References

(1) Wu, M.; Benckiser, E.; Haverkort, M. W.; Frano, A.; Lu, Y.; Nwankwo, U.; Brück, S.; Audehm, P.; Goering, E.; Macke, S.; Hinkov, V.; Wochner, P.; Christiani, G.; Heinze, S.; Logvenov, G.; Habermeier, H.-U.; Keimer, B. Strain and Composition Dependence of Orbital Polarization in Nickel Oxide Superlattices. *Phys. Rev. B* **2013**, *88* (12), 125124. https://doi.org/10.1103/PhysRevB.88.125124.

(2) Disa, A. S.; Kumah, D. P.; Malashevich, A.; Chen, H.; Arena, D. A.; Specht, E. D.; Ismail-Beigi, S.; Walker, F. J.; Ahn, C. H. Orbital Engineering in Symmetry-Breaking Polar Heterostructures. *Phys. Rev. Lett.* **2015**, *114* (2), 026801. https://doi.org/10.1103/PhysRevLett.114.026801.
